# A deep learning method for classification of HNSCC and HPV patients using single-cell transcriptomics

**DOI:** 10.3389/fmolb.2024.1395721

**Published:** 2024-05-30

**Authors:** Akanksha Jarwal, Anjali Dhall, Akanksha Arora, Sumeet Patiyal, Aman Srivastava, Gajendra P. S. Raghava

**Affiliations:** Department of Computational Biology, Indraprastha Institute of Information Technology, Delhi, India

**Keywords:** HNSCC, gene biomarkers, single cell transcriptomics, machine learning, deep learning, classification models

## Abstract

**Background:**

Head and Neck Squamous Cell Carcinoma (HNSCC) is the seventh most highly prevalent cancer type worldwide. Early detection of HNSCC is one of the important challenges in managing the treatment of the cancer patients. Existing techniques for detecting HNSCC are costly, expensive, and invasive in nature.

**Methods:**

In this study, we aimed to address this issue by developing classification models using machine learning and deep learning techniques, focusing on single-cell transcriptomics to distinguish between HNSCC and normal samples. Furthermore, we built models to classify HNSCC samples into HPV-positive (HPV+) and HPV-negative (HPV−) categories. In this study, we have used GSE181919 dataset, we have extracted 20 primary cancer (HNSCC) samples, and 9 normal tissues samples. The primary cancer samples contained 13 HPV− and 7 HPV+ samples. The models developed in this study have been trained on 80% of the dataset and validated on the remaining 20%. To develop an efficient model, we performed feature selection using mRMR method to shortlist a small number of genes from a plethora of genes. We also performed Gene Ontology (GO) enrichment analysis on the 100 shortlisted genes.

**Results:**

Artificial Neural Network based model trained on 100 genes outperformed the other classifiers with an AUROC of 0.91 for HNSCC classification for the validation set. The same algorithm achieved an AUROC of 0.83 for the classification of HPV+ and HPV− patients on the validation set. In GO enrichment analysis, it was found that most genes were involved in binding and catalytic activities.

**Conclusion:**

A software package has been developed in Python which allows users to identify HNSCC in patients along with their HPV status. It is available at https://webs.iiitd.edu.in/raghava/hnscpred/.

## 1 Introduction

Head and neck cancer, encompasses a variety of malignancies that affect the respiratory tract and upper digestive tract. Head and Neck Squamous Cell Carcinoma (HNSCC) is the most typical kind among the head and neck cancer (Mody et al., 2021). In 2020, 562,328 people were diagnosed with head and neck cancer (HNC) worldwide, with a total count of 277,587 deaths due to the disease (Broutian et al., 2020). These carcinomas often develop in the salivary glands, larynx, oral cavity, throat, and sino-nasal tract epithelium. A number of head and neck malignancies are linked to the human papillomavirus (HPV) infection, notably HPV-16. However, some malignancies are also related to the other carcinogens like smoking, excessive alcohol, and other factors depending on the country or area. Hence, we can classify this cancer into two major categories—HPV-negative and HPV-positive. The median age of diagnosis for HPV associated HNSCC is about 66 years, whereas for HPV-associated oropharyngeal cancer the median age is ∼53 years (Johnson et al., 2020).

**FIGURE 1:**
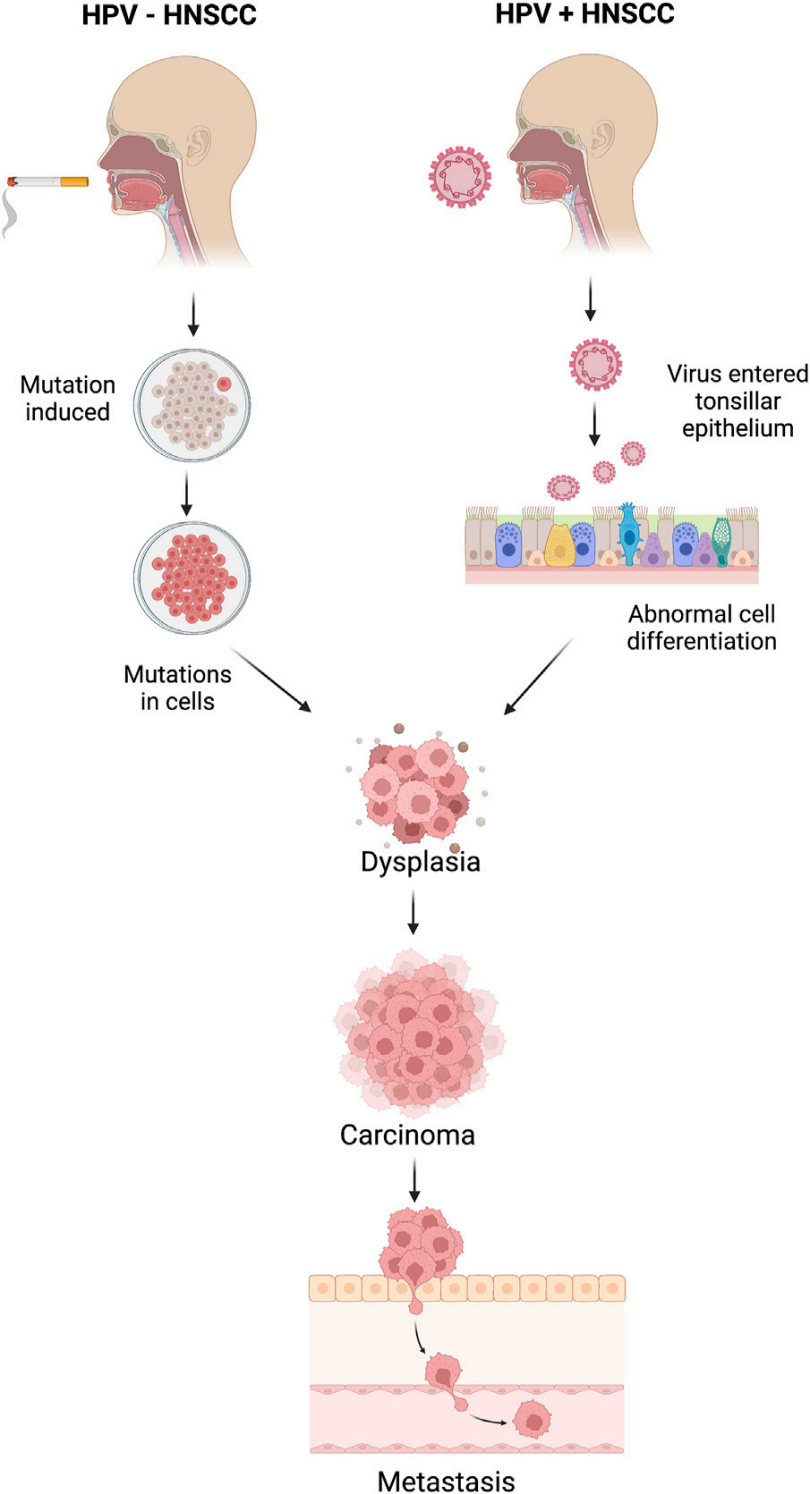
Mechanisms of head and neck squamous cell carcinoma (HNSCC) for HPV-positive and HPV-negative HNSCC patients.

Distinguishing between HPV-positive and HPV-negative Head and Neck Squamous Cell Carcinoma (HNSCC) samples holds profound significance in clinical practice as it unveils distinct molecular mechanisms underlying tumorigenesis and guides tailored therapeutic interventions. HPV-positive HNSCCs, primarily driven by high-risk human papillomavirus (HPV) infection, often manifest with activated cell cycle pathways, particularly the retinoblastoma protein (pRB) pathway, leading to enhanced cell proliferation (Leemans et al., 2018). Conversely, HPV-negative tumors frequently arise from genomic instability induced by environmental factors such as tobacco and alcohol exposure, resulting in diverse genetic alterations, such as mutations in tumor suppressor genes and oncogenes. Consequently, HPV-positive tumors exhibit heightened sensitivity to radiotherapy and chemotherapy due to their intact DNA repair mechanisms and increased expression of apoptosis-regulating proteins (Fakhry et al., 2008). Conversely, HPV-negative tumors, characterized by aberrant DNA repair pathways and resistance to apoptosis, necessitate more aggressive therapeutic strategies. Understanding the HPV status in HNSCC thus facilitates personalized treatment approaches, optimizing patient outcomes by targeting specific molecular vulnerabilities (Dok and Nuyts, 2016). Better understanding the HPV status of HNSCC tumors enables clinicians to tailor treatment strategies and provide accurate prognostic information, ultimately improving patient management and outcomes (Ang et al., 2010; Chaturvedi et al., 2011; Gillison et al., 2012). The mechanisms of HPV+ and HPV- associated HNSCC are explained in Figure 1.

Despite thorough and targeted treatment efforts, the chances of survival are reduced due to the majority of head and neck cancer cases being diagnosed at advanced stages. The traditional diagnosis of HNSCC is based on the physical examination, radiological investigation, and histological analysis of the tissue sections obtained from biopsies or surgical resections. These procedures can take a lot of time and are susceptible to mistakes in observation or interpretation, which can lead to discrepancies in cancer grading and prognostication (Mahmood et al., 2021). In addition to this, most of the HNSCC cancers are detected at a later stage. The reasons range from limited symptomatology in early-stage patients, swift progression from early to advanced stage, indistinctive diagnostic characteristics, and imprecise history information (Basheeth and Patil, 2019).

Identification of molecular biomarkers of HNSCC can lead to early diagnosis of this cancer and can also help in preventive management of HNSCC. The cancer biomarkers not only influence diagnosis but they also have the potential to improve the treatment outcomes using targeted therapy. The currently known biomarker of HNSCC is PD-L1 which is commonly used in treatment decision making in advanced stage of HNSCC. It has a moderate predictive value and has many limitations due to the lack of standardization and highly dynamic nature of PD-L1 expression. Currently, there are no any other FDA approved molecular biomarkers for HNSCC diagnosis or prognosis (Basheeth and Patil, 2019).

**FIGURE 2:**
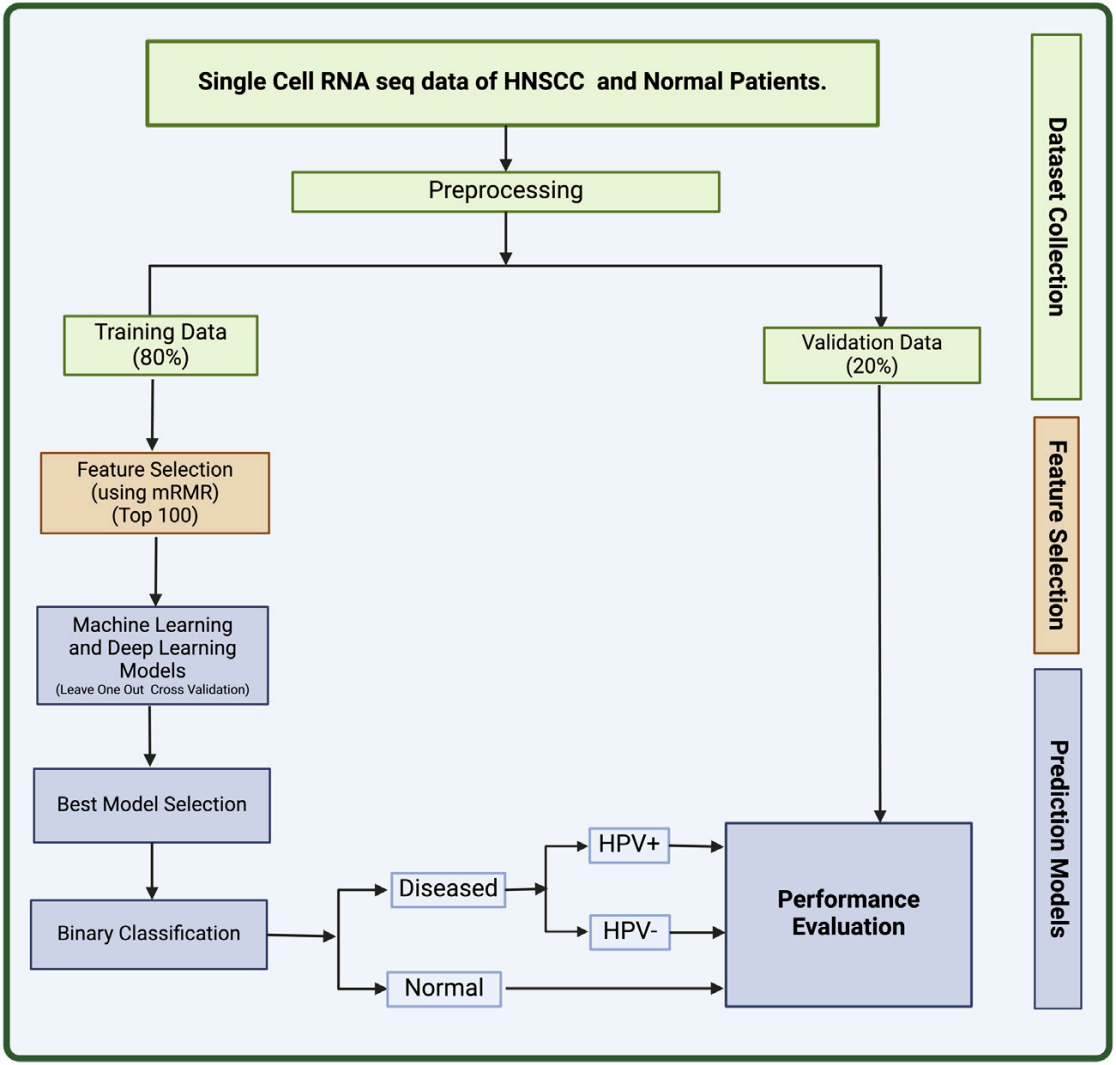
The full workflow of the study.

In this study, we made an attempt to identify biomarkers for HNSCC using single-cell sequencing data. On the basis of the 100 biomarkers identified in this study, we have developed a method that can predict the HNSCC cancer along with HPV+ or HPV− status. Single-cell data collected from individual cells using next-generation sequencing methods provides a better knowledge of the activity of a single cell in relation to its microenvironment (Eberwine et al., 2014). Cell-to-cell variation can be revealed by single-cell sequencing of RNA or epigenetic alterations, which may aid the populations in quickly adapting to new circumstances (Saliba et al., 2014). The significance of gene mosaicism, as well as intra-tumor genetic heterogeneity in the genesis of cancer or response to therapy, can be uncovered by single-cell precision (Gawad et al., 2016). Single-cell technology makes it possible to detect molecular alterations in individual cancer cells. This can increase the research of more specialized biomarkers with excellent resolution, leading to the development of a complete landscape of distinct cell types within tumors (Radpour and Forouharkhou, 2018). The full workflow of this study is described in Figure 2.

## 2 Materials and methods

### 2.1 Data collection

We retrieved the dataset used in this study (GSE181919) from National Centre for Biotechnology Information’s (NCBI) Gene Expression Omnibus (GEO) (Clough and Barrett, 2016; Choi et al., 2023). The GSE181919 dataset comprise of 37 tissue specimens from 23 patients with Head and Neck Squamous Cell Carcinoma (HNSCC), covering a range of tissues, including normal tissues (*n* = 9), precancerous leukoplakia (*n* = 4), primary HNSCC (*n* = 20), and metastasized tumors (*n* = 4). Choi et al. methodology involved aligning sequencing data to the human reference genome (GRCh38) and processing it using CellRanger 2.1.1 by 10X Genomics. Subsequently, cell-level transcripts were clustered using the “Seurat”package’s shared nearest neighbor method. To ensure the clarity in the dataset, we chose two distinct groups: normal tissue (*n* = 9) and primary HNSCC tissues (*n* = 20). Therefore, in this study, we have only taken 29 total samples comprising 20 primary cancer samples, and 9 normal samples. In addition, these cancer samples are divided into 13 HPV− and 7 HPV+ samples. The information on whether the samples were derived from HPV+ or HPV− patients was derived from the metadata provided on GEO. This dataset used Illumina HiSeq 4000 as the platform for scRNA sequencing. The 80% of this dataset was used to train machine learning (ML) and deep learning (DL) models and 20% was used as validation set.

### 2.2 Data pre-processing

After the retrieval of data from GEO, we processed the data using in-house python scripts. Firstly, we converted the sparse data into a matrix and removed insignificant columns from our training data. The genes that had no mapped readings to more than 80% of the cells were eliminated, and cells containing zeroes were filtered leading to 2,604 genes. The sequencing depth affects the range of values for the features, which necessitates normalizing the count data before doing any sort of analysis. Hence, we performed counts per million (CPM) normalization and log transformation on the data using scanpy package in python (Wolf et al., 2018).

### 2.3 Feature selection

We applied feature selection to the set of 2,604 genes obtained after pre-processing to obtain a set of biomarkers for HNSCC. This was achieved using mRMR (Minimum Redundancy and Maximum Relevance) feature selection algorithm (Radovic et al., 2017). mRMR selects a subset of features that have the least correlation amongst themselves but high correlation with the output class. The advantage of using this method is that it provides with a small set of features with high predictive potential. The redundancy between genes is taken into account in this technique in addition to the relationship between samples and genes. The most relevant feature will be considered out of the numerous identical features. We used the value K = 100 for mRMR to extract 100 most relevant genes for the prediction of HNSCC (Zhao et al., 2019a). This strategy has been previously demonstrated to be useful and often utilized in single-cell RNA sequencing analysis (Ding and Peng, 2005; Radovic et al., 2017).

### 2.4 Top dysregulated genes

After extracting the top 100 genes from feature selection, we performed a T-test analysis for the mean expression of genes in the cells of the groups Normal vs. Cancer and Cancer HPV+ vs. Cancer HPV−. We also wished to identify the top most dysregulated genes in both the comparisons. To achieve this, we found mean difference between the two classes in both comparisons (Normal vs. Cancer and Cancer HPV+ vs. Cancer HPV−), and reported the 10 most dysregulated (5 upregulated and 5 downregulated) genes with the highest difference in means for each comparison.

### 2.5 Machine learning models

We have developed various machine learning (ML) models to classify between normal subjects and HNSCC patients. In addition, we have also classified HNSCC patients into HPV positive and HPV negative. These machine learning models include Extreme Gradient Boosting (XGB), Decision Tree (DT), K-Nearest Neighbors (KNN), Extra Trees (ET), Logistic Regression (LR), and Random Forest (RF) algorithms. Hyperparameter tuning was also used to optimise the parameters of these algorithms. The DT classifier is a supervised machine learning model that classifies the output by learning decision rules from input, the KNN classifier predicts on the basis of the maximum number of votes cast in support of the class that is closest to the nearest neighbouring data point, LR classifier uses a logistic function to calculate the likelihood of an event, XGB Classifier is a distributed gradient-boosted decision tree machine learning package that offers simultaneous tree boosting, and RF classifier trains a number of decision trees to produce a single tree. A technique for ensemble supervised machine learning that makes use of decision trees is called extra trees. (Breiman, 2001; Wu et al., 2002; Geurts et al., 2006; Stoltzfus, 2011; Bulac and Bulac, 2016; Chen and Guestrin, 2016). These methods have previously been used in many studies (Aggarwal et al., 2023; Arora et al., 2023; Kaur et al., 2023; Srivastava et al., 2023).

### 2.6 Deep learning models

Along with the ML models, we have also applied deep learning classification technique—Artificial Neural Network (ANN) to classify the data (Wang, 2003). In this method, networks are composed of multiple layers, and each layer has a number of nodes (or neurons) that support decision making. The model architecture of ANN used in this study includes three hidden layers and an output layer. A dropout of 0.5 is implemented at each step to lessen the overfitting by neural network. Biological neuron networks served as the basis for this strategy. Artificial neurons, which are constructed from a network of connected units or nodes and are conceptually similar to the neurons in the human brain, are used to build ANNs. They consist of several layers, and inside each layer there are multiple nodes (or neurons) that support decision-making. The anticipated label (Diseased or Normal) of the sample is the final result. The final result classifies the samples into HNSCC positive or negative, and if found HNSCC positive then whether the patient is HPV positive or negative is identified.

### 2.7 Cross validation

The dataset was primarily composed of training data, which made up 80% of it and validation set, which made up the remaining 20%. In the LOOCV (Leave One out Cross Validation) approach, the whole training set is separated into N equivalent folds using the LOOCV technique, with (N-1) being utilized for training and the single fold being used for testing. Each fold serves as testing data for the technique’s N iterations. The overall performance was calculated as the mean of N iterations. This is a common practice in many types of studies (Peng et al., 2015; Vabalas et al., 2019).

### 2.8 Evaluation parameters

To evaluate the efficacy of various prediction models, we employed a number of evaluation indicators. In this study, we used both threshold-independent and threshold-dependent parameters. To calculate threshold-dependent characteristics like sensitivity (Sens), specificity (Spec), precision, F1-Score, and accuracy (Acc), we utilised the following formulae. We also used the conventional threshold-independent parameter Area Under the Curve (AUC) to assess the performance of the models. The metrics calculated in this study are mentioned in Eqs 1–5.

$$Sensitivity=\frac{P_{t}}{P_{t}+N_{f}}$$ (1)

$$Specificity=\frac{N_{t}}{N_{t}+P_{f}}$$ (2)

$$Accuracy=\frac{P_{t}+N_{f}}{P_{t}+N_{t}+P_{f}+N_{f}}$$ (3)

$$F1‐score=\frac{2P_{t}}{2P_{t}+P_{f}+N_{f}}$$ (4)

$$Precision=\frac{P_{t}}{P_{t}+P_{f}}$$ (5)

Where, *P*_*t*_ is true positive, *N*_*t*_ is true negative, *P*_*f*_ is false positive, and *N*_*f*_ is false negative.

## 3 Results

### 3.1 Feature selection

We applied a feature selection technique called mRMR to obtain a list of highly relevant features (genes) for the detection of HNSCC samples from a set of 2,604 genes that were obtained after data pre-processing (Zhao et al., 2019b). We obtained a subset of 100 genes that were able to classify HNSCC and non-HNSCC samples as well as HPV+ and HPV− samples correctly.

### 3.2 Top dysregulated genes

After performing T-test on selected 100 genes for Normal vs. Cancer and Cancer HPV+ vs. Cancer HPV− groups. It was found that all the genes were significantly differentially expressed with *p*-values<0.05 in Normal vs. Cancer comparison whereas 94 genes out of 100 were significantly differentially expressed with *p*-values<0.05 in Cancer HPV+ vs. Cancer HPV− comparison. The list of selected 100 genes along with their *p*-values, mean gene expressions, mean expression difference, and t-statistics for Normal vs. Cancer and Cancer HPV+ vs. Cancer HPV− are given in Supplementary Tables S1, S2, respectively. We also identified the top 10 dysregulated genes (5 upregulated and 5 downregulated) on the basis of mean difference between two classes in both the comparisons. The top 10 dysregulated genes for Normal vs. Cancer and Cancer HPV+ vs. Cancer HPV− are given in Tables 1, 2, respectively.

**TABLE 1:** Top 10 dysregulated genes for Normal vs. Cancer.

Gene	Mean gene expression cancer	Mean gene expression normal	Mean difference (cancer-normal)	T-Statistic	*p*-value	Up/Downregulated
CFD	0.773	57.497	−56.724	−85.985	0.000e+00	Downregulated
DCN	4.840	35.536	−30.696	−72.891	0.000e+00	Downregulated
GSN	2.566	29.935	−27.369	−76.183	0.000e+00	Downregulated
MGP	0.707	13.759	−13.052	−56.266	0.000e+00	Downregulated
MFAP4	0.206	7.802	−7.596	−82.346	0.000e+00	Downregulated
RPL28	63.478	15.950	47.528	103.250	0.000e+00	Upregulated
EEF1A1	85.286	35.414	49.872	82.304	0.000e+00	Upregulated
RPS19	77.935	17.512	60.423	92.379	0.000e+00	Upregulated
RPLP1	111.729	30.115	81.614	95.136	0.000e+00	Upregulated
B2M	158.129	43.524	114.605	107.234	0.000e+00	Upregulated

**TABLE 2:** Top 10 dysregulated genes for Cancer HPV- vs. Cancer HPV+.

Gene	Mean gene expression cancer	Mean gene expression normal	Mean difference (cancer-normal)	T-Statistic	*p*-value	Up/Downregulated
B2M	112.908	190.234	−77.326	−40.719	0	Downregulated
HLA-B	27.504	52.285	−24.781	−38.936	0	Downregulated
HLA-A	27.495	47.94	−20.445	−32.086	1.01E-220	Downregulated
HLA-C	25.502	40.151	−14.649	−29.336	1.27E-185	Downregulated
RPLP1	106.477	115.458	−8.981	−5.482	4.24E-08	Downregulated
CXCR4	6.556	5.289	1.267	10.654	1.95E-26	Upregulated
BTG1	12.936	11.385	1.551	9.614	7.81E-22	Upregulated
RPL28	64.482	62.765	1.717	1.887	0.0592	Upregulated
EEF1A1	86.865	84.166	2.699	2.439	0.0147	Upregulated
RPS19	80.110	76.391	3.719	2.797	0.0052	Upregulated

### 3.3 Model performance for HNSCC vs. non-HNSCC

We applied various ML models like DT, RF, ET, XGB, and KNN on our dataset, where we used 80% of dataset GSE181919 for training, 20% of dataset GSE181919 as validation set, and. It was observed that machine learning models were able to achieve an AUROC of 0.85 (XGB, ET) on the validation set. In order to increase the AUROC, we applied DL algorithm—ANN on the dataset and observed that the AUROCs increased to 0.91 for the validation set. The complete results for the ML and DL performances are given in Table 3.

**TABLE 3:** Performance of ML and DL models for the classification of HNSCC patients and normal subjects.

Training set							
Models	Accuracy	MCC	AUROC	Sensitivity	Specificity	Precision	F1 score
Decision Tree	0.93	0.85	0.93	0.95	0.91	0.94	0.94
Random Forest	0.96	0.92	0.96	0.98	0.94	0.96	0.97
Logistic Regression	0.92	0.84	0.92	0.95	0.88	0.92	0.94
XGB	0.92	0.83	0.92	0.92	0.92	0.95	0.93
ExtraTree	0.97	0.80	0.90	0.91	0.89	0.91	0.91
KNN	0.93	0.85	0.92	0.96	0.89	0.93	0.94
Deep Learning Model	0.99	0.93	0.97	0.98	0.96	0.97	0.98

Validation Set							
Models	Accuracy	MCC	AUROC	Sensitivity	Specificity	Precision	F1 Score
Decision Tree	0.85	0.70	0.83	0.97	0.70	0.80	0.88
Random Forest	0.85	0.71	0.83	0.99	0.68	0.79	0.88
Logistic Regression	0.79	0.60	0.77	0.98	0.56	0.73	0.84
XGB	0.86	0.73	0.85	0.98	0.71	0.81	0.89
ExtraTree	0.86	0.74	0.85	0.99	0.71	0.81	0.89
KNN	0.83	0.68	0.81	0.98	0.65	0.77	0.87
Deep Learning Model	0.92	0.82	0.91	0.94	0.89	0.94	0.94

### 3.4 Model performance for HPV+ vs. HPV−

After classification of samples as HNSCC or non-HNSCC, we attempted to classify whether an HNSCC sample belonged to an HPV+ or HPV− class. Hence, we developed ML and DL models to further classify the HNSCC samples as HPV+ and HPV−. The maximum AUROC achieved by ML models was 0.81 (XGB) for the validation set. After employing ANN classifier to the data, it was observed that the AUROC increased to 0.84 for the validation set. The results for HPV+ and HPV− classification from HNSCC patients are summarized in Table 4.

**TABLE 4:** Performance of ML and DL models for the classification of HPV+ and HPV− patients from HNSCC patients.

Training set							
Models	Accuracy	MCC	AUROC	F1 score	Sensitivity	Specificity	Precision
Decision Tree	0.75	0.50	0.75	0.78	0.79	0.71	0.77
Random Forest	0.82	0.63	0.81	0.84	0.87	0.75	0.81
Logistic Regression	0.84	0.67	0.84	0.86	0.86	0.82	0.85
XGB	0.77	0.52	0.76	0.79	0.81	0.71	0.77
ExtraTree	0.84	0.68	0.84	0.86	0.88	0.8	0.84
KNN	0.80	0.59	0.79	0.82	0.84	0.75	0.8
Deep Learning Model	0.991	0.980	0.995	0.992	0.989	0.993	0.995

Validation Set							
Models	Accuracy	MCC	AUROC	F1 score	Sensitivity	Specificity	Precision
Decision Tree	0.69	0.35	0.65	0.74	0.76	0.59	0.72
Random Forest	0.84	0.88	0.80	0.83	0.91	0.92	0.94
Logistic Regression	0.80	0.54	0.75	0.85	0.9	0.61	0.81
XGBClassifier	0.82	0.61	0.81	0.86	0.87	0.74	0.86
ExtraTree Classifier	0.46	−0.11	0.76	0.52	0.58	0.33	0.47
K Neighbours classsifier	0.49	−0.06	0.55	0.55	0.58	0.38	0.52
Deep Learning Model	0.84	0.70	0.83	0.88	0.98	0.68	0.79

### 3.5 Gene ontology

**FIGURE 3:**
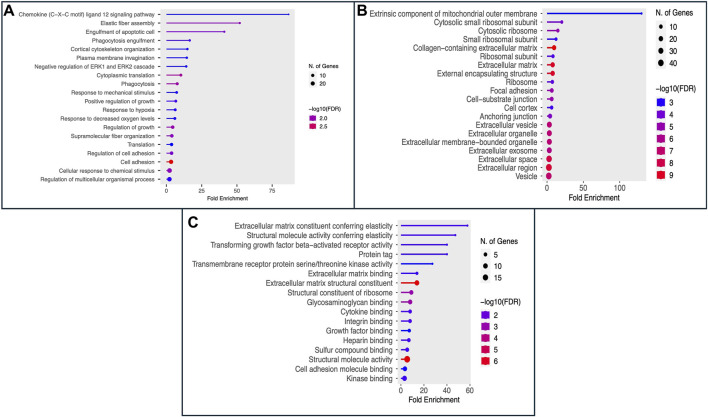
The figure displays the Gene Ontology (GO) enrichment analysis results as **(A)** Biological Process, **(B)** Cellular Component (CC), and **(C)** Molecular Function (MF) for the top 100 selected genes.

The Gene Ontology (GO) encapsulates our understanding of the biological world in three ways: molecular function, cellular component, and biological process (Ashburner et al., 2000; Gene Ontology Consortium, 2021). 100 genes that may serve as potential biomarkers of HNSCC were retrieved once mRMR analysis was complete. On these 100 retrieved genes, we next ran Gene Ontology (GO) enrichment analysis using PantherDB to map the biological processes, cellular components, and molecular functions of the chosen genes (Mi et al., 2013). The findings of the GO enrichment analysis for all 100 selected genes are displayed in Supplementary Tables S3–S5, respectively. The results of Gene Ontology for Biological Processes and Cellular Component are shown in Figure 3A, B respectively. We see that the majority of genes have a role in the binding activities of many metabolic processes as shown in Figure 3C. The genes and their roles are described in Figure 3.

## 4 Discussions

One of the heterogeneous diseases, HNSCC affects the head and neck region, namely the oral cavity, paranasal sinuses, larynx, nasal cavity, hypopharynx, and oropharynx. It is described by malignant and uncontrollable cell proliferation in these locations (Hsieh et al., 2019). Advancement in the sequence technology allows the researchers to find various biomarkers such as diagnostic, predictive, and prognostic biomarkers. These biomarkers help in better understanding of the disease as well as may aids in designing novel and effective diagnosis and treatment. A biomarker is described as a biological molecule present in the blood, other body fluids, as well as in tissues, that serves as a sign of a normal or aberrant process, a condition, or a disease by the National Cancer Institute (NCI). To determine how effectively the body will react to an illness or condition medication, a biomarker could well be utilized (Hsieh et al., 2019). This study aims to find out a set of potential biomarkers from single-cell transcriptomic data of head and neck cancer patients that can classify HNSCC patients and normal individuals with reliable accuracy. In addition, we have also attempted to classify HNSCC patients as HPV+ or HPV−. The biomarkers identified in this study could aid in the early diagnosis and screening of HNSCC.

To categorize non-cancer and HNSCC disease cells from their single-cell RNA seq data, we employed a variety of machine learning models, including an ANN deep learning model. We also further tried to categorize the diseased patients into HPV+ and HPV−. We trained the model with 80% of the dataset GSE181919, 20% of the dataset GSE181919 as validation set. The datasets were originally quite extensive and had a significant number of features. During the preprocessing step, the feature count was decreased to a shallow level of 2,604 genes (features). One of the feature selection techniques known as mRMR was used to obtain the limited set of features which could be helpful in categorizing the samples because many characteristics were correlated. The top 100 genes with the least amount of redundancy and the most relevance were extracted from these 2,604 genes (features) using mRMR. Furthermore, 100 genes (features) separated the HNSCC patients from non-cancer with an accuracy of around 92%, an AUROC of 0.91 in the validation set. Whereas in the case of HPV classification, the metrics obtained were, AUROC 0.83% and 98% accuracy on the validation set. For the detection and categorization of biomarkers, ANN has proven to be an effective technique among all machine learning models.

After obtaining the top 100 most relevant genes for the classification of HNSCC, we performed Gene Ontology (GO) enrichment analysis using PantherDB and most of the genes were observed to be related to catalytic and binding activities (Mi et al., 2013). Some of them also had a role in other essential processes like ATP-dependent activity, molecular function regulator, molecular transducer, structural molecule activity, translation regulator activity, transcription regulator, and transporter activity. Many of the genes identified in this study have been previously linked to HNSCC in earlier studies. The gene PLAC9’s overexpression has been reported in connection with the inhibition of cell growth regulation and has also been reported in connection with cancers such as ovarian cancer and breast cancers as prognostic biomarkers (Ouyang et al., 2018). Gene “ACKR1”, along with other 3 genes in a study, was reported to be downregulated in HNSCC patients, which was correlated with poor prognosis (*p* < 0.05) (Liu et al., 2022). Also, gene “AQP7,” which is involved in physiologically functional cell migration, was upregulated in MSR of patients with ten tumors (Zivicova et al., 2018). Whereas, gene FXYD1 was reported to be downregulated in the cancer samples, while FXYD4 and FXYD5 were overexpressed (*p* < 0.05, fold change>1.5) (Jin et al., 2021). In a study on cancer cells, it was observed that BTG1 gene overexpression was linked to tumor growth or lung metastasis, inhibited proliferation, and induced differentiation in different types of cancer cells (Zhao et al., 2020). Also, mutations occurring in different genes, including B2M, CDKN2A, is found to be related with the occurrence and development of tumors in Head and neck cancer patients (Wang et al., 2020). As shown in the study Sun et al., 2020, genes such as MFAP4, CD37, CXCL12, ADH1B, SOD3, SCARA5, ANGPTL1, FHL1, F10, CXCR4, MEG3, TXNIP, GDF10, and ABI3BP are downregulated in head and neck squamous cell carcinoma as they operate as potential tumor suppressor genes, inhibiting tumor cell proliferation, invasion, and migration while also promoting apoptosis (Sun et al., 2020). By controlling the expression of miR-421 and E-cadherin, MEG3 long-encoding RNA inhibits the development of head and neck squamous cell carcinoma. However, additional research into MEG3’s downstream mechanism in controlling the molecular process of epithelial-mesenchymal transformation (EMT) in head and neck squamous cell carcinoma (HNSCC) development is required (Ji et al., 2020). Growth differentiation factor-10 (GDF10), also known as BMP3b, is a tumor suppressor that belongs to the transforming growth factor-b (TGF-b) superfamily (Cheng et al., 2016). CIB1, PIM3, SLC16A3, VOPP1, BMP4, TIGIT, ADAR, and LRRN4CL are studied as upregulated genes in various cancer types such as squamous carcinoma cells, breast cancer, head and neck squamous cell carcinoma, and pancreatic cancer (Baras et al., 2011; Alarmo et al., 2013; Zheng and Tian, 2014; de Jong et al., 2018; Notarangelo, 2018; Broutian et al., 2020; Yu et al., 2020; Huo et al., 2021; Wen et al., 2021; Yang et al., 2022). A complex that is important in the keratinocyte-intrinsic immune response to human papillomaviruses (-HPVs) is formed when CIB1 interacts with the EVER1, and EVER2 proteins (de Jong et al., 2018; Notarangelo, 2018). It has been observed that nearly all primary HNSCCs express at least one PIM kinase member at high levels (Broutian et al., 2020). Immunological checkpoint T cell immunoreceptor with immunoglobulin and ITIM domain (TIGIT) is essential for immune suppression. However, it has a connection to genetics and epigenetics, and a role in tumor immunity (Wen et al., 2021). The transforming growth factor (TGF) superfamily includes extracellular signaling molecules known as bone morphogenetic proteins (BMPs), which are known to control cell proliferation, differentiation, and motility, particularly during development. Functional research shows that, particularly in HNSCC cancer, has connected BMP4 to the encouragement of cell migration and the suppression of cell proliferation (Alarmo et al., 2013).

Overall, most of the genes which were obtained from our study have been reported as promising candidate for biomarkers in various studies (Zivicova et al., 2018; Broutian et al., 2020; Sun et al., 2020; Wang et al., 2020; Zhao et al., 2020; Jin et al., 2021; Liu et al., 2022). However, some genes have not yet been reported in connection with Head and Neck cancer. These genes may require further investigation and study. These genes may act as novel findings which could help in diagnose patients with Head and neck cancer. In order to help the scientific community, we created a Python package called “HNSCPred” based on the aforementioned work (https://webs.iiitd.edu.in/raghava/hnscpred/). To fully understand how the discovered genes impact and contribute to the progression of HNSCC disease, further clinical investigations on these genes are necessary.

## Data Availability

The datasets presented in this study can be found in online repositories. The names of the repository/repositories and accession number(s) can be found below: https://webs.iiitd.edu.in/raghava/hnscpred/.
